# Patterns of malpractice claims and compensation after surgical procedures: a retrospective analysis of 8,901 claims from the Finnish patient insurance registry

**DOI:** 10.1186/s13037-023-00353-0

**Published:** 2023-02-10

**Authors:** Maiju Welling, Annika Takala

**Affiliations:** 1 The Finnish Patient Insurance Center, 00084 Vakuutuskeskus, P.O. Box 1, Helsinki, Finland; 2 University of Helsinki and Helsinki University Hospital, 00029 HUS, P.O. Box 705, Helsinki, Finland

**Keywords:** Claim, Claims, Patient injury, Patient safety, Surgery, Insurance-based, No-fault, No-blame, Liability

## Abstract

**Background:**

Invasive surgical procedures carry risk of harm to patients. In addition to avoidable harm, disparities between patient expectations and the outcome of a procedure may lead to patient injury claims. The follow-up of claims and compensation is an important entity for patient safety. The number of claims should be related to the surgical volume, so that a healthcare provider can benchmark with similar organizations and see if its trends are developing favourably or deteriorating. Our objective was to find out the claims and compensation rates due to surgery in an insurance-based system.

**Methods:**

Data related to surgical claims and reference volume in the period 2011–2015 were collected from the claim register of the Finnish Patient Insurance Centre and benchmarking community register of Finnish operating departments. The data included age, gender, hospital, year of surgery, surgical code, and outcome of the claim.

**Results:**

There were 8,901 claims related to the corresponding reference group of 1,470,435 surgical procedures. The claims rate was 0.61% and compensation rate was 0.22%. Trends for claims and compensation rates decreased over the study period. In high volume procedures, a low compensation rate was detected for excision of tonsils and adenoids, Caesarean section and extracapsular cataract operations using the phacoemulsification technique. A high compensation rate was detected for primary prosthetic replacement of the hip and knee joints and decompression of spinal cord and nerve roots. Unreasonable injury (death or permanent deterioration of health) was compensated in 2.4 per 100,000 procedures.

**Conclusions:**

Register data research in a no-fault patient insurance system revealed a claims rate of 6 per 1,000 procedures and compensation rate of 2 per 1,000 procedures. A decreasing trend in both rates over the study period was detected. Different surgical procedures exhibit varying claims and compensation rates.

**Supplementary Information:**

The online version contains supplementary material available at 10.1186/s13037-023-00353-0.

## Introduction

Patient harm is a global concern. Conservative assessments have deemed unsafe care to be the 14th leading cause of morbidity and mortality in the world [1]. Medical treatments always include some risk, but any preventable harm that occurs is too much. Large international reviews have estimated that around 10% of hospital admissions lead to an adverse event, and half of these adverse events are preventable [2]. According to a more recent meta-analysis, one in every 20 patients suffers from preventable harm in medical care, the incidence being highest in intensive care and surgery [3]. Focusing patient safety efforts on preventable harm has proven to be an efficient way to improve patient safety [4], but the first step is to know what our current state of safety is. A better understanding of adverse events in different healthcare settings is vital for quality and safety improvement [5].

Healthcare is an extremely complex environment where people interact with each other and a wide range of devices and technologies [6]. Safety in complex sociotechnical systems is best understood when examined through the systems approach; it acknowledges that human errors are inevitable. Therefore, adverse events need to be identified and investigated in a way that allows the root causes of the events to be corrected [2]. This requires a no-blame culture in which safety concerns can be addressed without asking who caused the harm but rather why it happened.

Closed claims analyses produce information that is associated with ultimate failure in healthcare. Systems that handle the litigation process of malpractice differ. Nordic countries have no-fault patient insurance systems, which aim to foster no-blame safety culture. In no-fault systems, compensation is not based on negligence. An ideal system for compensating medical injuries encourages healthcare providers to report errors, enhances quality improvement efforts, increases openness in the patient–physician relationship, and yet enables corrective action towards a healthcare provider when needed [7].

Surgical providers carry a risk of preventable adverse events [8]. Patient injury claims made by hip and knee replacement patients in Finland have been found to correlate with revisions and infections, implying that claims can be used as a quality indicator [9]. In order to use claims for this purpose, the number of claims needs to be adjusted by the volume of treatment provided. In Finland, this has been done earlier for some specialties or procedures [9–13]. Previous studies suggest that claims and compensation rates vary considerably, but the rates across all surgical procedures are unknown. Identifying procedures or organizations with exceptionally high claims and compensation rates can expedite investigations into the causes of higher-than-average risk and lead to improved safety [14].

This study aimed to reveal the claims and compensation rates and their annual trends related to a large surgical procedure cohort in Finland. The study also evaluated differences in claims and compensation rates between different procedures, patient groups and hospitals.

## Methods

In 1987, Finland became the first country to deploy a statutory patient insurance covering all healthcare provided nationwide. The basic principles of patient insurance have remained unchanged over the years. All healthcare providers in Finland are obliged to have patient insurance. Compensation may be paid for bodily injuries sustained by patients in connection with healthcare and medical treatment given in Finland [15]. Patients may claim compensation by filing a notice of injury to the Finnish Patient Insurance Centre (PIC), which is responsible for centralized handling of claims. The patient must file the claim within three years of the date that he/she became aware of the possible injury. The PIC then obtains all necessary clarifications, including patient documents, from the relevant healthcare provides. Experienced medical experts evaluate these cases, and juridical experts are consulted when necessary.

The patient insurance legislation includes several compensation criteria. Most often, compensation is paid based on the preventability of the injury. If an experienced professional would likely have avoided the injury by acting in a different way, the injury is compensated as a so-called treatment injury. Severe infections and unreasonable injuries can be compensated even if they were not preventable. An injury may be compensable as unreasonable if it has led to permanent severe illness, injury, or death, and it is materially disproportionate with the initial situation. Another compensation criterium that is relevant for surgical procedures concerns equipment-related injuries, which covers surgical instruments and other devices used to perform procedures.

The statistical compilation of surgical procedures in Finland is based on the codes of The Nordic Medico-Statistical Committee (NOMESCO) classification of surgical procedures. The classification is slightly nationally modified and is maintained by the Finnish Institute of Health and Welfare.

The data for this study were collected from two registers retrospectively and consisted of surgical procedures from the period 2011–2015. Patient injury cases with reference to surgical procedure were obtained from the claim register of the PIC. The data comprise age, gender, hospital, year of surgery, surgical code, and outcome of the claim. The reference data for the total extent of surgical procedures were obtained from Benchmarking register of Finnish operating departments (BM-OR®, provided by Tieto-Evry, Helsinki, Finland). The data set included age group or gender (different data sets), hospital, operating department, surgical code, and year of surgery. The age groups were under 1 year, 1 to 5 years, 6 to 15 years, 16 to 44 years, 45 to 64 years, 65 to 74 years, and 75 years or over.

During the study period there were 36 operating departments in 28 different hospitals voluntarily participating in BM-OR® for at least some of study period. All the university hospitals in Finland (*n* = 5) are members of BM-OR®. We analysed the PIC data against the corresponding data of BM-OR® (reference group).

SPSS 25.0 software was used to run statistical analyses. Proportions were compared with a Pearson χ2 test and proportional trends with a Mantel–Haenszel test of linear association as appropriate. A *p* value of < 0.05 was considered statistically significant.

This study was conducted according to the tenets of the Declaration of Helsinki. The PIC and the steering board of BM-OR® accepted the study plan. The study did not involve human participants. All data provided were anonymous without any personal identifiable information. Ethical approval and informed consent were not required.

## Results

**Fig. 1:**
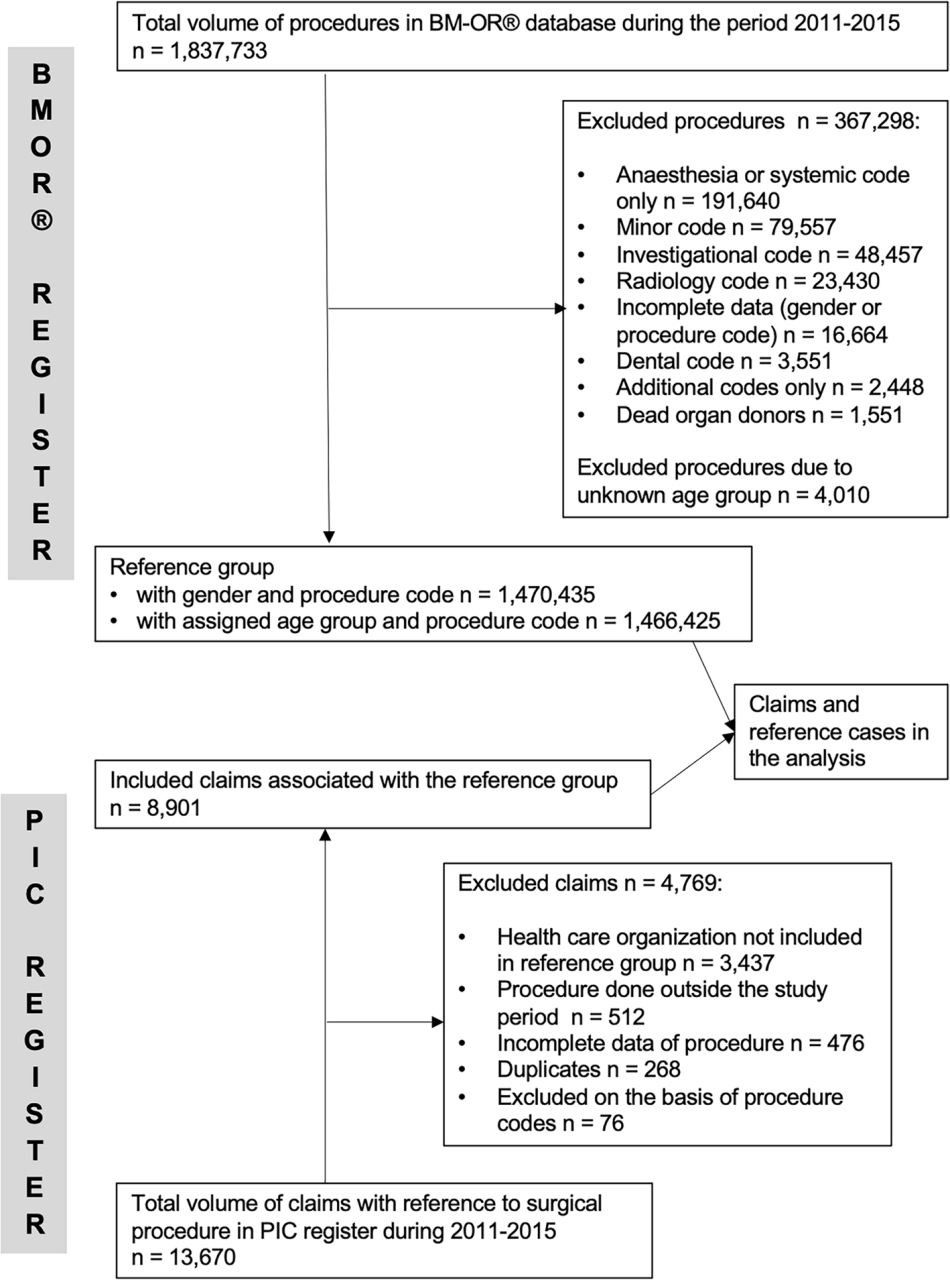
The combination of register data for analysis. BM-OR®; benchmarking community of Finnish operating departments. PIC; Finnish Patient Insurance Centre

Data for 1,837,733 procedures were collected from the BM-OR® database during the period 2011–2015. We excluded 367,298 procedures, namely anaesthesia codes, additional codes, investigational procedures, radiological codes, dental codes, codes for death organ donors, and cases with incomplete data (Fig. 1). Thus, the reference group consisted of 1,470,435 cases (811,715 women, 658,720 men). Data on age group were available for 1,466,425 cases (Fig. 1). The numbers and claims rates of the most frequent 20 procedures in the reference group are presented in Table 1.

**Table 1:** The number and claims rate (%) of the 20 most frequent surgical procedures

**Procedure**	**Number**^**a**^	**Claims rate**^**b**^ (%)
Extracapsular cataract operations using phacoemulsification technique	87,261	0.33
Caesarean section	41,566	0.38
Primary prosthetic replacement of hip joint	39,357	1.82
Excision of tonsils and adenoids	39,229	0.05
Repair of inguinal hernia	39,057	0.29
Operations on gallbladder	32,395	0.54
Operations for impaired function of peripheral nerves	31,784	0.44
Primary prosthetic replacement of knee joint	31,233	1.59
Appendectomy	28,910	0.38
Decompression of spinal cord and nerve roots	25,624	1.60
Surgery of eardrum and middle ear	20,705	0
Total excision of uterus	19,814	1.08
Intraocular operations on vitreous body and retina	19,200	0.52
Fracture surgery of femur	19,006	1.03
Fracture surgery of ankle and foot	18,375	1.21
Partial excision of mammary gland	18,019	0.28
Excision, reconstruction, and fusion of joints of ankle and foot	16,846	1.82
Partial excision of prostate	16,545	0.34
Partial excision of intestine	16,218	1.12
Excision and repair of lesion of skin of head and neck	15,895	0.16

^a^Total number of procedures presented in the reference (BM-OR®) group during the period 2011–2015

^b^Respective claims during the period 2011–2015 presented in the Finnish Patient Insurance (PIC) register related to total number of procedures

PIC data comprised 13,670 claims related to surgical procedures during the period 2011–2015. The number of claims applied to the reference group was 8,901 (Fig. 1). The overall claims rate was 0.61%. Compensation was awarded in 3,206 cases, and the compensation rate was 0.22%. There were 960 different surgical codes presented in the reference group, whereas PIC data indicated claims for 441 different codes. The grounds for PIC decisions are shown in Table 2.

**Table 2:** Grounds for PIC decisions

Compensated patient injuries related to	Number of cases (% of all compensated cases)
treatment	2,834 (88.39)
infection	317 (9.89)
equipment	16 (0.50)
accident	3 (0.10)
unreasonable outcome	36 (1.12)
total	3,206 (100)
Compensation denied because injury was	Number of cases (% of all denials)
unavoidable or tolerable	4,248 (74.59)
not related to treatment	1,399 (24.57)
minor	21 (0.37)
not covered by patient insurance	27 (0.47)
total	5,695 (100)

*PIC* Finnish Patient Insurance Centre

**Fig. 2:**
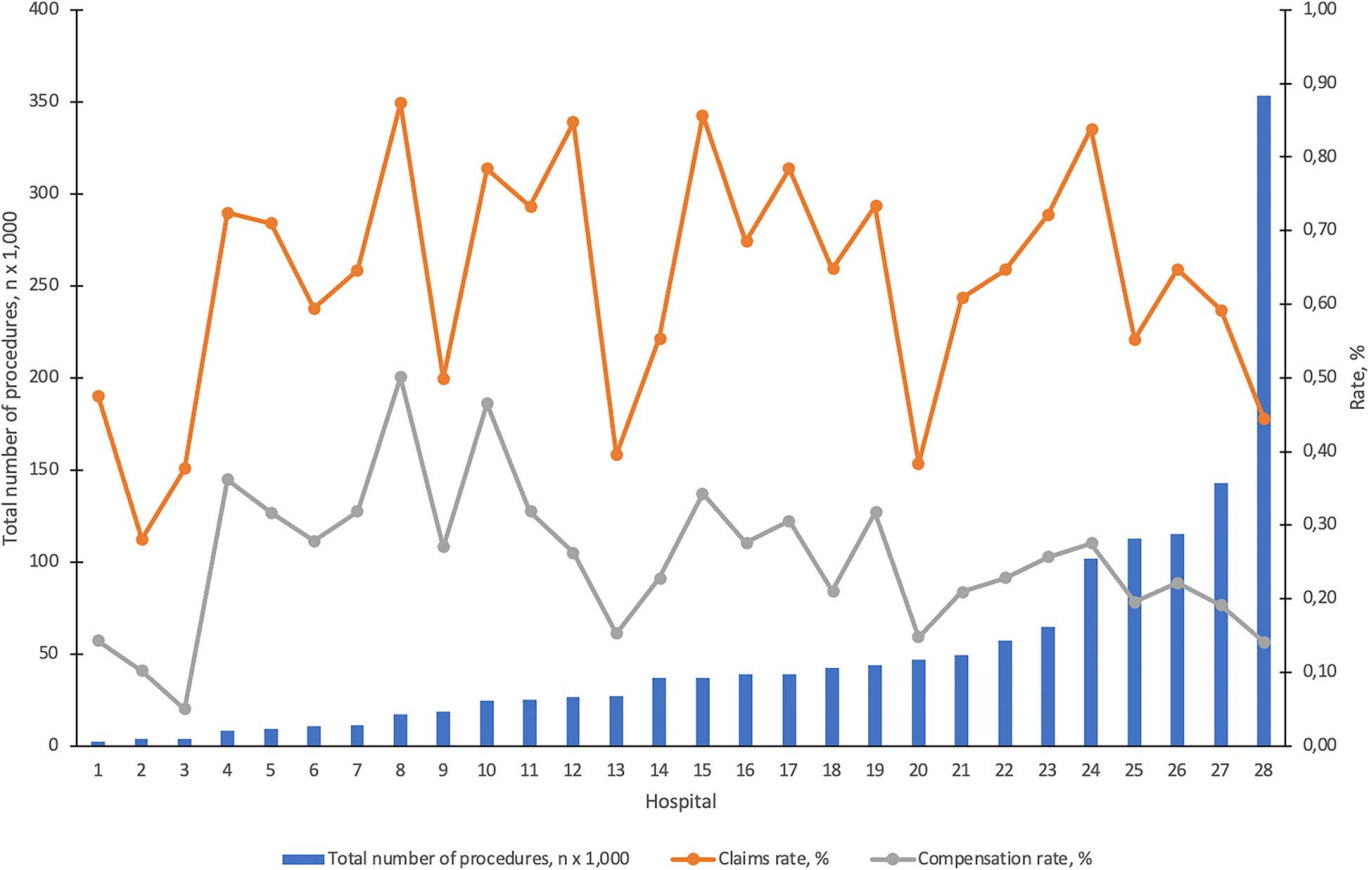
The total number (sum of included data during the period 2011–2015, histogram) of surgical procedures, with claims (orange line) and compensation (grey line) rates in each hospital

The rate of claims and compensation showed a decreasing trend from 0.64% and 0.25% in 2011 to 0.56% and 0.19% in 2015, respectively (*p* ≤ 0.001, Table 3). The share of compensated claims out of all claims (compensated claims rate) varied annually from 38.09% in 2011 to 34.34% in 2014. The total number of surgical procedures and rates of claims and compensation during the study period in the reference group hospitals are presented in Fig. 2. Neither claims rate nor compensation rate correlated significantly with the total number of procedures in each hospital (*r* = -0.127 and -0.261, respectively). The number of claims and total number of procedures per hospital correlated with each other significantly (*r* = 0.965, *p* < 0.01).

**Table 3:** Annual numbers of claims, compensated claims and surgical procedures with respective rates during the period 2011–2015

Year	Claims (n)	Comp. claims (n)	Procedures (n)	Claims rate (%)^a^	Compensation rate (%)^b^	Comp. claims rate (%)^c^
2011	1,675	638	259,827	0.64	0.25	38.09
2012	1,902	703	303,955	0.63	0.23	36.96
2013	1,816	659	297,315	0.61	0.22	36.29
2014	1,814	623	308,041	0.59	0.20	34.34
2015	1,694	583	301,297	0.56	0.19	34.42
All	8,901	3,206	1,470,435	0.61	0.22	36.02

*n* Number of cases

*Comp.* Compensated

^a^Pearson Chi-Square *p* = 0.001 and Mantel–Haenszel test of linear association *p* < 0.001

^b^Pearson Chi-Square and Mantel–Haenszel test of linear association *p* < 0.001

^c^Mantel–Haenszel test of linear association *p* = 0.006

The proportion of females among claimants and compensated cases was significantly higher compared to that of males (0.65% and 0.23% vs. 0.55% and 0.20%, respectively, *p* < 0.001). Among claimants, however, the compensation was equally distributed between men and women. There was a statistically significant difference between age groups when comparing claims rate, compensation rate and compensated claims rate (all *p* < 0.001, Table 4). The highest claims rate and compensation rate were detected in the age group 45 to 64 years, whereas the compensated claims rate was highest in the age group 6 to 15 years.

**Table 4:** Claims rate, compensation rate and compensated claims rate in different age groups during the period 2011–2015

Age group in years	Claims (n)	Comp. claims (n)	Procedures (n)	Claims rate** (%)	Compensation rate** (%)	Comp. claims rate**(%)
< 1	63	17	9,549	0.66	0.18	26.98
1–5	40	21	47,033	0.09	0.04	52.50
6–15	121	68	58,859	0.21	0.12	56.20
16–44	1,910	667	373,174	0.51	0.18	34.92
45–64	3,681	1,337	457,633	0.80	0.29	36.32
65–74	1,899	679	265,865	0.71	0.26	35.76
> 75	1,187	417	254,312	0.47	0.16	35.13
All	8,901	3,206	1,466,425	0.61	0.22	36.02

*n* Number of cases

*Comp.* Compensated

^**^Pearson Chi-Square test *p* < 0.001

During the five-year period, there was a total of 120 surgical codes that had more than 10 claims and more than 100 procedures done. For procedures with over 20,000 cases as reference (Table 1) the claims rate was low for surgery of the eardrum and middle ear and for excision of the tonsils and adenoids. Regard to procedures with over 20,000 cases, the compensation rate was low for excision of the tonsils and adenoids (compensation rate 0.02%), Caesarean Sect. (0.06%) and extracapsular cataract operations using the phacoemulsification technique (0.08%).

There were 50 surgical codes with a claims rate above 1% (see Additional file 1). The primary prosthetic replacement of ankle and foot joints had a claims rate of 18%, but only 300 such procedures were done over the study period. With a reference number above 20,000 procedures, primary prosthetic replacement of hip (claims rate 1.82%) and knee (1.59%) joints and decompression of the spinal cord and nerve roots (1.60%) presented high claims rates. The procedures with a reference number above 20,000 and a high compensation rate were primary prosthetic replacement of the hip joint (compensation rate 0.8%), decompression of the spinal cord and nerve roots (0.7%) and primary prosthetic replacement of the knee joint (0.6%).

The compensated claims rate was above 60% for operations on bones of the elbow and forearm (81.25%), exteriorization of the intestine and creation of intestinal stomas (69.23%), fracture surgery of the wrist and hand (65.82%), and operations on adhesions in intestinal obstruction (61.54%). The highest compensated claims rate of those procedures with a reference number above 20,000 were operations for impaired function of peripheral nerves (compensated claims rate 46.10%), primary prosthetic replacement of the hip joint (43.93%), decompression of the spinal cord and nerve roots (43.90%), and operations on the gallbladder (43.68%). Operations for intracranial aneurysms and other vascular lesions, aorto-coronary venous bypass, thromboendarterectomy of the femoral artery and branches, and glaucoma filtering operations had compensated claims rates of less than 10% (range 0–9.09%). Caesarean Sect. (16.67%) had the lowest compensated claims rate of those procedures with a reference number above 20, 000.

Unreasonable injury was compensated in 36 cases (2.44 per 100,000 procedures). There were five procedures with multiple unreasonable injuries: excision, reconstruction, and fusion of the spine (4 cases, rate for unreasonable injury 0.028%), decompression of the spinal cord and nerve roots (3 cases, 0.012%), primary prosthetic replacement of the hip joint (3 cases, 0.008%), operations for impaired function of peripheral nerves (2 cases, 0.006%), and bariatric operations on the stomach (2 cases, 0.053%). Most patients with unreasonable injury were adults (35 cases) and one was below 1 year. Unreasonable injury was not detected in age groups 1 to 5 years or 6 to 15 years. The annual rate of unreasonable injuries varied from 1.3 per 100,000 (year 2015) to 3.7 per 100,000 (year 2013), but the rates did not differ statistically between the years.

## Discussion

Research on closed claims and patient injuries has highlighted important safety and quality aspects of surgical procedures. Analyses concerning proportion of malpractice and patient injury data related to surgical procedures are infrequent. This study investigated patient injury claims and compensation rates in a large Finnish cohort of registered surgical procedures. Differences in claims and compensation rates between different procedures, patient groups and hospitals were also evaluated. The results show that approximately 6 out of 1,000 patients who had undergone a surgical procedure filed a patient injury claim. Two patients per 1,000 received fiscal compensation. The claims and compensation rates over the study period showed a downward trend. There was variation in the claims and compensation rates between different surgical procedures. Neither claims nor compensation rates had statistically significant correlations with the volume of procedures in the hospital. The claims rate may be a useful indicator for healthcare organizations to follow.

The Finnish patient insurance system covers all healthcare provided in Finland. Filing a notice of injury to the PIC is free of charge. Patients or next of kin can easily file a notice without juridical assistance. The system fosters a no-blame patient safety culture. Individual healthcare professionals are not liable to compensate for injuries. Good coverage of patient insurance makes the claim register of the PIC a valuable data source for research. Only a few countries in the world collect similar data on patient injury claims.

The overall claims rate has been previously studied in the Swedish patient insurance system, which closely resembles that of Finland. In the Swedish study, the claims rate during the period 1997–2004 for surgical specialties was 0.36% and compensation was paid in 35 to 67% of claims [14]. Both studies recognize primary prosthetic replacement of the hip and knee joints and decompression of peripheral nerves as prone to high claims rates. Respectively, both studies show that a high number of claims is detected with high-volume procedures such as extracapsular cataract operations and Caesarean sections, but their compensation rates are low. Thus, reliable evaluation of surgical safety and quality should be based on indicators proportional to total volume. Unfortunately, this kind of analysis is still quite rare.

Claims and compensation rates both exhibited continuous annual downward trends in our study. The worldwide focus on surgical safety was promoted by a surgical safety checklist [16] just prior the study period. The awareness of patient safety issues in general and among surgical specialties has developed favourably. This may have also boosted the decline in avoidable patient injuries [17]. The occurrence of unavoidable serious injuries was low in the present study suggesting the most severe outcome of patient injury happens sporadically.

The compensated claims rate varied between procedures from 0 to 81.25% in the present study. We suggest that compensated claims rate reflects the congruence between patient experience and physician review. A low compensated claims rate may be due to surgery failing to fulfil a patient’s expectations of an outcome. This highlights that, in addition to surgical performance, the outcome of treatment is dependent on the trust between a patient and the healthcare system. Communication about adverse events beforehand, patient education [18, 19], and proper rehabilitation [20–22] improve the quality of surgical care. A patient-reported outcome measurement after surgery should be part of standard data collection in order to further develop the quality of invasive procedures [23].

The true economic burden of patient injuries is difficult to assess. Whatever the compensation system (insurance or juridical), a healthcare organization should be prepared for the possible financial impact of injuries. The healthcare organization as a “third victim” and the accompanying consequences may lead to defensive medicine and increase costs of care [24]. The extent of compensation varies according to the outcome of the injury [25]. The loss of income and some other compensation may be paid for several years or decades after the occurrence of a patient injury. The mathematical mean of compensation paid by the PIC in surgical patient injuries varied from 11,624–13,779 euros during the period 2011–2015. The annual cost of compensation in Finland is approximately 40 million euros for a population of 5.5 million. In addition to the direct costs of patient insurance, patient injuries cause notable costs to healthcare organizations in the form of additional procedures, lengthened treatment times, and resources needed to handle the paperwork and psychological support needed. Based on the volume and types of surgery, a healthcare organization should approximate the resources needed for liability. Acknowledging the costs of injuries should help to steer more effort and funding to preventive safety work.

Different countries have different systems for handling medical malpractice. Finland has a no-fault patient insurance system in which compensation is not based on negligence. The methodology of the present study can be used in different systems and settings. The main idea is that the number of claims and amount of compensation should be related to the surgical volume to enable benchmarking. Following rates instead of absolute numbers is also beneficial for recognition of temporal trends.

The results of this study show differences in patient injury frequencies for different surgical procedures. These findings should help professionals worldwide to recognize and pay attention to possible high-risk procedures and their proper risk assessment. More research is needed to evaluate whether injuries accumulate in similar procedures in different countries.

This study reveals the claims and compensation rates for surgical patient injuries, but the number of patient injuries that were not reported is not known. Despite the ease of filing a notice of injury, some patients never do. Not all patients are aware of their rights or capable of defending them. However, according to the Act on the Status and Rights of Patients (785/1992), all healthcare providers in Finland must have a patient ombudsman, who advises and helps to file notices of injury. It is, however, likely that the majority of essential patient injuries are included in the register of the PIC.

There may be issues that partly explain differences in claims rates and compensation rates between different procedures. Some patient groups may be more aware of their rights than others. For example, there is an active osteoarthritis patient society in Finland, which informs its members on filing a notice of injury. Also, changes in surgical techniques may affect that contents of claims reveal less severe harm, although the overall claims rate for an individual procedure seems to be on a steady level.

The process from surgery to claim and decision may take years. When considering an individual surgical procedure, improvements must originate from more immediate and sensitive patient-reported outcome measures. This is achieved by quality and research projects. Thus, we must consider that the register data provide a view on the past, and the current situation may be somehow different.

The reported low compensation rate for Caesarean section needs to be interpreted with caution. This study did not include data on natural deliveries. The claims and compensation rates for Caesarean sections should be compared with corresponding rates of vaginal deliveries, not with claims and compensation rates of other surgical procedures. We highlight that the results of this study should not be used to assess the safety of deliveries.

Neither claims rates nor compensation rates were found to significantly correlate with the volume of procedures in an individual hospital. The register data used in the study did not include patient characteristics other than age (or age group) and gender. Therefore, it is not possible to evaluate the differences in the risk levels of surgical procedures performed in different hospitals. Some rare or otherwise exceptionally demanding procedures are centralized to large university hospitals. On the other hand, patients of smaller hospitals in sparsely populated areas are, on average, older and have more chronic conditions than patients of hospitals in densely populated areas. To properly analyse the correlation between operational volume of hospitals and patient injury claims or compensation, the risk level of the surgical procedures should be standardized.

## Conclusion

The results of this study highlight the importance of relating patient injury data to volume. In this register data research, the claims rate was 6 per 1,000 procedures and compensation rate 2 per 1,000 procedures, but there were differences in the claims and compensation rates for different surgical procedures. Over the study period, claims and compensation rates both showed downward trends, suggesting an improvement of patient safety in surgery over time. In order to reduce patient injuries, more attention should be paid to those procedures with exceptionally high compensation rates. Also, high-volume procedures with normal to high compensation rates need to be focused on.

## Supplementary Information

**Additional file 1.** The list of 50 procedures that had a reference number over 100 procedures, at least 10 claims and claims rate above 1 %.

## Data Availability

The data is available on reasonable request.

## Abbreviations

PIC: The Finnish Patient Insurance Centre
NOMESCO: The Nordic Medico-Statistical Committee
BM-OR®: Benchmarking register for Finnish operating departments

## References

[1] Jha AK, Larizgoitia I, Audera-Lopez C, Prasopa-Plaizier N, Waters H, Bates DW (2013). The global burden of unsafe medical care: analytic modelling of observational studies. BMJ Qual Saf.

[2] Rafter N, Hickey A, Condell S, Conroy R, O'Connor P, Vaughan D (2015). Adverse events in healthcare: learning from mistakes. QJM.

[3] Panagioti M, Khan K, Keers RN, Abuzour A, Phipps D, Kontopantelis E (2019). Prevalence, severity, and nature of preventable patient harm across medical care settings: systematic review and meta-analysis. BMJ.

[4] Pronovost PJ, Colantuoni E (2009). Measuring preventable harm: helping science keep pace with policy. JAMA.

[5] Jonsson PM, Øvretveit J (2008). Patient claims and complaints data for improving patient safety. Int J Health Care Qual Assur.

[6] Carayon P, Bass E, Bellandi T, Gurses A, Hallbeck S, Mollo V (2011). Socio-technical systems analysis in health care: A research agenda. IIE Trans Healthc Syst Eng.

[7] Studdert DM, Brennan TA (2001). No-Fault Compensation for Medical Injuries: The Prospect for Error Prevention. JAMA.

[8] de Vries EN, Ramrattan MA, Smorenburg SM, Gouma DJ, Boermeester MA (2008). The incidence and nature of in-hospital adverse events: a systematic review. Qual Saf Health Care.

[9] Järvelin J, Häkkinen U (2012). Can patient injury claims be utilised as a quality indicator?. Health Policy.

[10] Kouhia S, Vironen J, Hakala T, Paajanen H (2015). Open Mesh Repair for Inguinal Hernia is Safer than Laparoscopic Repair or Open Non-mesh Repair: A Nationwide Registry Study of Complications. World J Surg.

[11] Järvelin J, Rosenqvist G, Häkkinen U, Sintonen H (2009). Patient and hospital characteristics associated with claims and compensations for patient injuries in coronary artery bypass grafting in Finland. J Health Serv Res Policy.

[12] Helkamaa T, Hirvensalo E, Huhtala H, Remes V (2016). Patient injuries in primary total hip replacement. Acta Orthop.

[13] Blomgren K, Aaltonen L-M, Lehtonen L, Helmiö P (2018). Patient injuries in operative rhinology during a ten-year period: Review of national patient insurance charts. Clin Otolaryngol.

[14] Pukk-Härenstam K, Ask J, Brommels M, Thor J, Penaloza RV, Gaffney FA (2009). Analysis of 23364 patient-generated, physician-reviewed malpractice claims from a non-tort, blame-free, national patient insurance system: lessons learned from Sweden. Postgrad Med J.

[15] Mikkonen M (2004). Prevention of patient injuries: the Finnish patient insurance scheme. Med Law.

[16] Haynes A, Weiser T, Berry W, Lipsitz SR, Breizat A-HS, Dellingeret EP (2009). A Surgical Safety Checklist to Reduce Morbidity and Mortality in a Global Population. N Engl J Med.

[17] Harrison W, Narayan B, Newton A (2015). Litigation costs of wrong-site surgery and other non-technical errors in orthopaedic operating theatres. Ann R Coll Surg Engl.

[18] Yin B, Goldsmith L, Gambardella R (2015). Web-Based Education Prior to Knee Arthroscopy Enhances Informed Consent and Patient Knowledge Recall: A Prospective, Randomized Controlled Study. J Bone Joint Surg Am.

[19] Shahmoradi L, Rezaei N, Rezayi S, Zolfaghari M, Manafi B (2022). Educational approaches for patients with heart surgery: a systematic review of main features and effects. BMC Cardiovasc Disord.

[20] Di Monaco M, Vallero F, Tappero R, Cavanna A (2009). Rehabilitation after total hip arthroplasty: a systemic review of controlled trials on physical exercise program. Eur J Phys Rehabil Med.

[21] Guerra ML, Singh PJ, Taylor NF (2015). Early mobilization of patients who have had a hip or knee joint replacement reduces length of stay in hospital: a systematic review. Clin Rehabil.

[22] Kokotovic D, Berkfors A, Gögenur I, Ekeloef S, Burcharth J (2021). The effect of postoperative respiratory and mobilization interventions on postoperative complications following abdominal surgery: a systematic review and meta-analysis. Eur J Trauma Emerg Surg.

[23] Ortega G, Allar BG, Kaur MN, Edelen MO, Witt EE, Fayanju OM (2022). Prioritizing Health Equity in Patient-reported Outcome Measurement to Improve Surgical Care. Ann Surg.

[24] Mira J, Lorenzo S, Carrillo I, Ferrús L, Pérez-Pérez P, Iglesias F (2015). Interventions in health organisations to reduce the impact of adverse events in second and third victims. BMC Health Serv Res.

[25] Vicente-Guijarro J, Valencia-Martín J, Fernández-Herreruela C, Sousa P, Solves JJM, Aranaz-Andréset JM (2022). Surgical Error Compensation Claims as a Patient Safety Indicator: Causes and Economic Consequences in the Murcia Health System, 2002 to 2018. J Patient Saf.

