# Supplementary material for: Patterns of malpractice claims and compensation after surgical procedures: a retrospective analysis of 8,901 claims from the Finnish patient insurance registry
Source: Patient Saf Surg. 2023 Feb 10;17:3. doi: 10.1186/s13037-023-00353-0 (PMC9912597; doi:10.1186/s13037-023-00353-0)
Supplement: Supplementary file 1 — Additional file 1. The list of 50 procedures that had a reference number over 100 procedures, at least 10 claims and claims rate above 1 %. [file 13037_2023_353_MOESM1_ESM.pdf]

Additional file 1. The list of 50 procedures that had a reference number over 100 procedures, at least 10 claims and claims rate above 1 % (n;number, %;percent, comp;compensated).

Procedure	Claims (n)	Comp. cases (n)	Procedures (n)	Claims rate (%)	Compensation rate (%)	Comp. claims rate (%)
Primary prosthetic replacement of ankle and foot joints	54	20	300	18.00	6.67	37.04
Operations on vertebrae	18	7	468	3.85	1.50	38.89
Local operations on intestine	32	11	1,010	3.17	1.09	34.38
Operations on bone of knee and lower leg	37	15	1,517	2.44	0.99	40.54
Resection of oesophagus	12	3	508	2.36	0.59	25.00
Excision, reconstruction, and fusion of spine	333	140	14,452	2.30	0.97	42.04
Operations on bone of elbow and forearm	16	13	696	2.30	1.87	81.25
Secondary prosthetic replacement of hip joint	132	44	6,017	2.19	0.73	33.33
Surgery of ascending aorta	27	3	1,252	2.16	0.24	11.11
Aorto-coronary venous bypass	18	1	898	2.00	0.11	5.56
Operations for gastro-oesophageal reflux disease	44	14	2,352	1.87	0.60	31.82
Excision, reconstruction, and fusion of ankle and foot joints	307	137	16,846	1.82	0.81	44.63
Primary prosthetic replacement of hip joint	717	315	39,357	1.82	0.80	43.93
Secondary prosthetic replacement of knee joint	47	12	2,736	1.72	0.44	25.53
Excision of rectum	75	30	4,386	1.71	0.68	40.00
Operations on tendons of hip and thigh muscles	30	11	1,803	1.66	0.61	36.67
Operations for aneurysm of infrarenal abdominal aorta and iliac arteries	23	6	1,396	1.65	0.43	26.09
Operations on capsules and ligaments of ankle and foot joints	11	4	680	1.62	0.59	36.36
Decompression of spinal cord and nerve roots	410	180	25,624	1.60	0.70	43.90
Primary prosthetic replacement of knee joint	496	186	31,233	1.59	0.60	37.50
Operations on muscles and tendons of knee and lower leg	16	9	1,022	1.57	0.88	56.25
Operations on bone of ankle and foot	146	64	9,586	1.52	0.67	43.84
Fracture surgery of knee and lower leg	151	51	10,064	1.50	0.51	33.77

Procedure	Claims (n)	Compensations (n)	Procedures (n)	Claims rate (%)	Compensation rate (%)	Comp. claims rate (%)
Operations for lesions of spinal cord and nerve roots	12	4	813	1.48	0.49	33.33
Incision, biopsy, resection, and repair of chest wall	15	8	1,047	1.43	0.76	53.33
Thyroid gland operations	144	48	10,186	1.41	0.47	33.33
Total colectomy	23	7	1,629	1.41	0.43	30.43
Operations on adhesions in intestinal obstruction	13	8	966	1.35	0.83	61.54
Operations on muscles and tendons of ankle and foot	73	22	5,581	1.31	0.39	30.14
Primary prosthetic replacement of shoulder	39	13	2,983	1.31	0.44	33.33
Excision, reconstruction, and fusion of wrist and hand	72	18	5,512	1.31	0.33	25.00
Partial excision or destruction of tumour of kidney and kidney pelvis	15	5	1,164	1.29	0.43	33.33
Fracture surgery of ankle and foot	223	81	18,375	1.21	0.44	36.32
Reconstructive operations on rectum	26	10	2,187	1.19	0.46	38.46
Fracture surgery of pelvis	12	4	1,012	1.19	0.40	33.33
Exploratory procedures on wrist and hand	17	7	1,487	1.14	0.47	41.18
Operations on fascia, ganglia, and bursae of knee and lower leg	17	6	1,497	1.14	0.40	35.29
Operations for intracranial aneurysm and other vascular lesions	26	0	2,312	1.12	0	0
Partial excision of intestine	181	66	16,218	1.12	0.41	36.46
Coronary artery bypass with internal mammary artery	87	13	7,827	1.11	0.17	14.94
Fracture surgery of shoulder and upper arm	113	51	10,332	1.09	0.49	45.13
Total excision of uterus	214	109	19,814	1.08	0.55	50.93
Fracture surgery of spine	23	6	2,208	1.04	0.27	26.09
Fracture surgery of femur	196	63	19,006	1.03	0.33	32.14
Operations on capsules and ligaments of knee	59	26	5,740	1.03	0.45	44.07
Repair of incisional hernia	62	28	6,057	1.02	0.46	45.16
Excision of pancreas	15	2	1,466	1.02	0.14	13.33
Bypass from femoral artery and branches	28	8	2,740	1.02	0.29	28.57

Procedure	Claims (n)	Compensations (n)	Procedures (n)	Claims rate (%)	Compensation rate (%)	Comp. claims rate (%)
Excision and destruction of intracranial lesion	63	11	6,286	1.00	0.17	17.46
Operations on capsules and ligaments of wrist and hand	22	13	2,210	1.00	0.59	59.09
